# The role of fire in UK peatland and moorland management: the need for informed, unbiased debate

**DOI:** 10.1098/rstb.2015.0342

**Published:** 2016-06-05

**Authors:** G. Matt Davies, Nicholas Kettridge, Cathelijne R. Stoof, Alan Gray, Davide Ascoli, Paulo M. Fernandes, Rob Marrs, Katherine A. Allen, Stefan H. Doerr, Gareth D. Clay, Julia McMorrow, Vigdis Vandvik

**Affiliations:** 1 School of Environment and Natural Resources, The Ohio State University, Kottman Hall, 2021 Coffey Road, Columbus, OH 43210, USA; 2 School of Geography, Earth and Environmental Sciences, University of Birmingham, Birmingham B31 2DX, UK; 3 Soil Geography and Landscape Group, Wageningen University, PO Box 47, Wageningen 6700 AA, The Netherlands; 4 NERC Centre for Ecology and Hydrology, Bush Estate, Penicuik, Edinburgh EH26 0QB, UK; 5 Department of Agricultural, Forest and Food Sciences, University of Torino, Largo Paolo Braccini 2, Grugliasco (TO) 10095, Italy; 6 Centro de Investigação e de Tecnologias Agro-Ambientais e Biológicas, Universidade de Tras-os-Montes e Alto Douro, Vila Real, Portugal; 7 School of Environmental Sciences, University of Liverpool, Liverpool L69 3GP, UK; 8 Department of Geography, Swansea University, Swansea, UK; 9 School of Environment, Education and Development, The University of Manchester, Manchester M13 9PL, UK; 10 Department of Biology, University of Bergen, Postboks 7803, Bergen 5020, Norway

**Keywords:** moorland, management burning, prescribed burning, peat, UK, *Calluna vulgaris*, wildfire

## Abstract

Fire has been used for centuries to generate and manage some of the UK's cultural landscapes. Despite its complex role in the ecology of UK peatlands and moorlands, there has been a trend of simplifying the narrative around burning to present it as an only ecologically damaging practice. That fire modifies peatland characteristics at a range of scales is clearly understood. Whether these changes are perceived as positive or negative depends upon how trade-offs are made between ecosystem services and the spatial and temporal scales of concern. Here we explore the complex interactions and trade-offs in peatland fire management, evaluating the benefits and costs of managed fire as they are currently understood. We highlight the need for (i) distinguishing between the impacts of fires occurring with differing severity and frequency, and (ii) improved characterization of ecosystem health that incorporates the response and recovery of peatlands to fire. We also explore how recent research has been contextualized within both scientific publications and the wider media and how this can influence non-specialist perceptions. We emphasize the need for an informed, unbiased debate on fire as an ecological management tool that is separated from other aspects of moorland management and from political and economic opinions.

This article is part of the themed issue ‘The interaction of fire and mankind’.

## 1. Introduction

**Figure 1.**
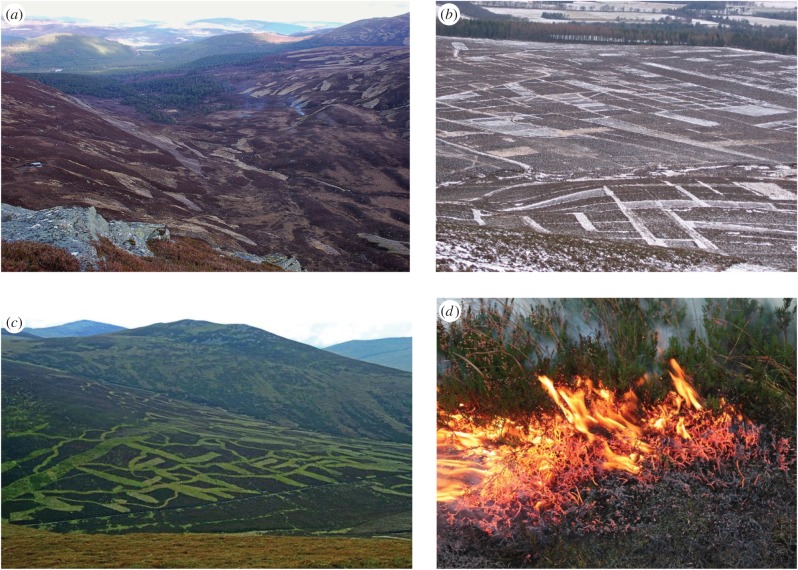
(*a–c*) Examples of moorlands managed through forms of prescribed burning typically associated with grouse moor management. Fire can, however, be used as an ecological tool for much more than just grouse and sheep production. Even grouse moor burning practice varies widely across the UK as can be seen here. Prescribed burning drives a variety of changes in peatland ecosystems including a range of ecosystem benefits and impacts according to the temporal and spatial scale one considers. Depending on how fires are managed, the ecological, visual and aesthetic impacts can be greater or lesser. All images were from geography.org.uk. (*d*) A low-severity prescribed burn moving through the lower canopy of a stand of *Calluna*, the moss and litter layer covering the peat surface is left more-or-less untouched. (Online version in colour.)

Fire, either as a management tool or as wildfire, is a landscape-scale disturbance and a critical regulator of the ecological, hydrological and biogeochemical function of landscapes around the world [1–4]. This is the case in UK upland landscapes that notably include large areas of peatland. British upland ecosystems are highly variable in character and cover a spectrum of abiotic and biotic conditions reflecting the north–south temperature and east–west moisture gradients across the country. Peatlands include dry heaths on thin peats with vegetation dominated by *Calluna vulgaris* (L.) Hull (hereafter referred to as *Calluna*); similar vegetation on thinner organic soils that, due to their shallow depth, are not officially recognized as peat; wet heaths on peat dominated by a mixtures of *Calluna*, *Erica tetralix* L., grasses, sedges and *Sphagnum* spp.; and blanket bogs on deep peat which have a mosaic of vegetation communities that include some dominated by *Sphagnum* spp., *Eriophorum* spp., *Molinia caerulea* (L.) Moench and other ericaceous species including *Calluna*. In the uplands, such ecosystems are often collectively referred to as ‘moorland’. While in some northern and western regions these ecosystems may have a natural origin (e.g. [5]), in most locations they are the result of forest clearance and domestic livestock grazing that may date back several thousand years [6]. Most moorland vegetation is highly flammable, which favoured the use of fire as an important tool in their management throughout the past [6]. Even apparently very wet bogs can be burnt in the early spring prior to the green-up of vegetation despite standing water at the ground surface [7]. Today, managed burning is strongly associated with habitat management for red grouse (*Lagopus lagopus scoticus* Latham 1787) on privately owned shooting estates. The current form of rotational patch burning associated with grouse moor management (figure 1) has been used for approximately the past 200 years [6]. However managed burning can be used to achieve a wide variety of ecological objectives and it is not only associated with traditional grouse moor management [8]. Fire was also an important component of the management of upland areas for cattle and sheep grazing prior to the popularization of grouse shooting (e.g. [9]) and its use may stretch back as far as the Mesolithic [10]. This parallels traditional land-use practices of similar antiquity throughout oceanic regions of northwest Europe, including Denmark [11], Norway [12] and Sweden [13]. That peatland ecosystems have long been modified by fire is thus widely recognized, indeed in some locations there is evidence that regular burning has occurred long enough to enhance the selection of fire-adaptive traits in peatland plant populations [14]. Against this historical context we now need to understand: (i) how the dynamic equilibrium that exists in all ecosystems subject to recurrent disturbance varies in response to different fire disturbance regimes; (ii) the extent to which differing fire regimes may drive changes in ecosystem state; and (iii) how ecosystem composition and state in turn affect the delivery of key ecosystem services.

Despite the complex, long-term role of fire in peatland management, there is a growing trend of simplifying the narrative around burning in the uplands of the UK. This can present managed burning as an ecological practice that is only ever damaging and responsible for the poor ecological condition of many heathland and peatland ecosystems. For example, the recent report by the Adaptation Sub-Committee [15] shows 27% of deep peat sites in England experience management burning but are not known to have been subject to any form of restoration action. Some media reports (e.g. [16]) have presented this as meaning that burning has destroyed 27% of England's blanket bogs. Recent studies have also identified the presence of burning on upland landscapes as being detrimental to these environments [17], or emphasize potentially negative consequences of burning particularly with regard to carbon (C) storage [18]. There are, however, also strong potential benefits from using fire as an ecological tool in oceanic heathlands and peatlands of Britain (e.g. [8,19]) and northwest Europe (e.g. [20,21]). The emphasis in both of the preceding sentences should be on *potential* because, overall, there is a paucity of evidence upon which to make informed decisions. Further, we find the way the debate is being framed is concerning; notably the lack of engagement with key concepts from fire ecology, and the sometimes provocative manner in which results are publicized. In our opinion, this reinforces the current, dominant narrative regarding burning in the UK, one which lacks nuance and appears to be influenced by political and economic conflicts as much as ecological understanding. Choices for future management are simplified into not burning (or even banning burning) versus continuing an intensive, stereotypical form of traditional rotational heather burning. In reality, existing practices are very spatially heterogeneous and the grouse moor stereotype (Figure 1) represents just one potential form of fire management. Many good, as well as poor, examples of practice exist. The tone of the debate makes completing much needed research problematic as land managers are less inclined to collaborate when the prevailing public perception of fire is negative and managers themselves can view scientists as having an agenda.

Here, based on recent peer-reviewed literature on the use of managed fire in the UK uplands, and its subsequent presentation in the wider media, we consider there is an urgent need for researchers to:

(1) Provide robust evidence of the interactions and trade-offs between the various practices associated with peatland management regimes (grazing, drainage, and fire).
(2) Consistently classify the effects of all vegetation fires according to fire severity. At its simplest level this means not confounding severe wildfire effects with those from management burns. Management fires are set in winter or early spring when soil heating is minimal. By contrast, wildfires predominantly occur in spring and summer during dry periods [22] when deep soil heating and peat ignition are much more probable. There is a continuum of burn severity across both managed burns and wildfires and this varies temporally and spatially [23–25].
(3) Develop appropriate guidelines for classifying peatland condition that account for their fire ecology.
(4) Generate informed and unbiased debate regarding peatland fire management that separates ecology from politics and economics.

## 2. Complexities in understanding the role of fire in peatland ecosystems

### (a) Interactions and trade-offs in peatland fire management

Any ecological disturbance has benefits and costs depending on the species or ecosystem in question. Where humans plan ecological disturbances for landscape management goals, it is essential to weigh up the trade-offs involved and make decisions that reflect the weighting given to different priorities. Debate over who should get to make such decisions, and how, is an important philosophical and political issue but is beyond the scope of this paper. In many ecosystems, fire is a natural process that plays a vital role in facilitating plant regeneration, improving forage quality and productivity, defining vegetation community composition, controlling landscape-scale variation in habitat structure, and modulating subsequent wildfire behaviour and severity (e.g. [1,2,26]). Managed fire superimposes or replaces natural fire regimes and reinforces ecological processes that depend on fire disturbance. Peatlands and moorlands in the UK are designated habitats, recognized for their conservation importance [27], but many are also cultural landscapes that owe their existence to the use of fire as a management tool [6]. Fire has long been, and still is, an integral part of the UK upland landscape. Effective landscape management that uses fire as a tool needs to use fire in a sustainable manner, integrating traditional approaches where appropriate, to maximize the desired ecosystem benefits or services and minimize disbenefits [3]. This will require an evidence-based approach adapted to suit local conditions, with some managed fire regimes better able to minimize trade-offs than others (no one size fits all). Previous authors (e.g. [8,19,27]) have suggested that such an approach could include: a reduction in the frequency of burning over blanket bog; a reduction in average burn size and increased variation in burn area to produce a more heterogeneous habitat mosaic; a reduction in the proportion of moorland burned and a greater amount of unburned heather; a shift in vegetation succession towards scrub on suitable parts of moorland (e.g. steep slopes) and maintaining fire-free buffers or restoring woodland around riparian systems. There is also evidence that burning may not be required to maintain *Calluna* productivity in all situations. Results from the study by MacDonald *et al.* [28] show that *Calluna* can regenerate by ‘layering’ and the formation of adventitious roots. This led to the recommendations that managers not burn stands which have not experienced fires in the last 40 years and which have well-developed heather layering; avoid burning *Calluna* in wet, shaded or humid situations where layering is likely; and concentrate burning activity where *Calluna* forms dense, continuous stands. While these management suggestions may seem like common sense, there remains surprisingly little scientific evidence to suggest what their outcomes would be in terms of patch or landscape-scale ecosystem structure, function and diversity.

It is not our aim here to provide an exhaustive review of the effects of fire on peatland environments or other ecosystems. Instead, we suggest readers refer to holistic reviews of the effect of fire on the environment [29] and specific reviews of the effects of fire on soils [30], peatland ecosystems [31,32], C and climate [33], human health [34], and UK moorlands [35]. It is, however, informative to draw attention to a number of recent, relevant studies that highlight the range of potential outcomes from burning. Within the diverse spectrum of fire effects, managed burning can have a range of potential benefits for peatland management, for example, removing dense canopies of *Calluna* via burning creates hydrological and light conditions that favour *Sphagnum* species over pleurocarpous mosses. Evidence from fire-prone black-spruce forested bogs in North America and mires in Sweden, for example, show that *Sphagnum* species are replaced by pleurocarpous mosses under dense canopies that can be removed by wildfire [36,37]. Experimental studies have shown that *Sphagnum* plants can regenerate from deeply buried stems [38], and in boreal systems *Sphagnum* plants have been observed to vigorously resprout following intense wildfires [37]. Reductions in *Calluna* canopy density are likely to be required to restore peat-forming vegetation on many degraded bogs, and fire may be an effective way to achieve this particularly if the *Calluna* is old and unlikely to resprout [39]. Evidence from the UK's only long-term replicated burning experiment shows positive effects of controlled burning as *Sphagnum* abundance was higher in 10-year burn rotations than in both 20-year rotations and locations that had not been burnt for approximately 90 years [40]. This result, perhaps surprising to some, is reinforced by other studies showing rapid recovery of *Sphagnum* populations following managed fires (e.g. [7,41]) and that, in laboratory experiments, even prolonged exposure to high temperatures can be followed by *Sphagnum* resprouting, i.e. high temperature alone does not kill the entire *Sphagnum* plant [41]. Based on this research, it appears hummock-forming species such as *Sphagnum capillifolium* (Ehrh.) Hedw, and *Sphagnum fuscum* (Schimp.) H. Klinggr are particularly resilient to fire, but data are needed on burn effects on other species.

Managed burning can have additional benefits and previous authors have documented the potential for a positive relationship between the use of fire and the diversity of vascular plants [42] and lichens [43], as well as populations of invertebrates [44] and other wildlife, though the relationships are often complex. For instance, Davies *et al.* [43] showed that post-fire trends in abundance differed between lichen species meaning the benefits of burning for diversity were recognized at the landscape scale due to the associated heterogeneity in stand ages. Bargmann *et al.* [45] noted similar results for carabid beetles but also showed particularly high alpha diversity in recently burnt stands. In the study by Tharme *et al.* [46], while red grouse and golden plover (*Pluvialis apricaria* L. 1758) populations were positively affected by prescribed burning, meadow pippits (*Anthus pratensis* L. 1758) were negatively impacted. Elsewhere in Europe, researchers have shown the benefit of prescribed fire use in preventing the loss of protected, internationally rare moorland ecosystems more generally (e.g. [47–50]), and in promoting seed regeneration and the diversity of ecologically and geographically restricted species [20,21]. Recent modelling work suggests that short-rotation prescribed moorland burning also minimizes direct C loss from combustion that could otherwise occur under a more severe wildfire regime [51]. Furthermore, burning can also produce substantial quantities of C in refractory forms, which contributes to the longer-term C sequestration potential of moorlands [52,53].

Against this backdrop of the potential positive effects of managed burning in the UK and elsewhere, the rhetoric against burning in the UK may seem odd. However, regular managed burning is also associated with negative impacts. These include evidence for altered stream water chemistry including increased dissolved organic carbon (DOC) production (e.g. [54]) that may indicate wider changes in C storage, which has financial implications for utility companies due to the need to treat coloration of drinking water from upland catchments. Ramchunder *et al.* [54] showed that streams draining catchments that were managed using burning contained increased particulate organic matter, suspended sediments, and aluminium, iron and DOC than unmanaged (non-burned) catchments. The differences in water quality were associated with major differences in benthic macroinvertebrate community structure. However, there is also contrary evidence on the production of DOC including that (i) prescribed burning is associated with changes in DOC quality and associated water coloration, rather than DOC quantity [55]; (ii) DOC is strongly associated with the dominance of *Calluna* rather than burning *per se* [56,57], and (iii) in Sweden (and elsewhere), increased coloration of water also occurs in areas without moorland burning and the levels could not be attributed to organic C alone [58,59]. There the prevailing hypothesis is that the coloration results from decreased acidification. Furthermore, as has been noted elsewhere [55,57] there is a disconnect between the direction and magnitude of DOC changes between plot-scale studies (e.g. [60]) and catchment-level monitoring (e.g. [61]). Further study is required to couple the processes between these two scales. Some authors have also questioned whether increased DOC transport offsite leads to net C loss or simply serves as a conveyer for some of it to be accumulated elsewhere [62]. In-stream degradation processes (e.g. photo-induced degradation, [63]) will also determine whether there will be a lag between export and the transfer of C to the atmosphere. Thus, it is likely that some prescribed burning regimes have an effect on DOC in some places, but the picture is far from simple.

Rates of peat accumulation have been noted to be lower in areas burnt by management fires [64,65], suggesting that in terms of C sequestration burning may not be beneficial. However, Garnett [64,65] examined only the shorter (10-year) burning rotation at the long-term Hard Hill experiment site (further described below) and thus the evidence may not be comparable with most prescribed burns on peatlands which typically occur at longer intervals. There are few complete C budgets from UK peatland sites subject to management burning, but some studies have indicated that managed fire may lead to an ‘avoided loss’ of C [66,67] where burnt plots are smaller sources of C than unburnt controls. However, at no time did the management interventions in those studies lead to a transition to a C sink. Evidence for the effects of fire on the microbial community are scarce and tend to come from wildfire studies rather than prescribed burning, but perturbation by fire may stimulate microbial activity within peat and increase the rate of decomposition [68] impacting C storage. The effects on the microbial community may also be persistent [69] and involve changes to methane oxidation processes [70] and substrate use by the soil microbial community [71]. Prescribed fire can also cause changes to soil temperature regimes [72,73] with likely effects on process such as peat respiration, methanogenesis and methanotrophy [74]. Taken alone the alterations to peat temperature regimes recorded by Grau *et al.* [72] would suggest likely increases in soil C fluxes. Although many studies have shown that peat temperature is a critical control on microbial activity (e.g. [75]), recent studies demonstrate that above- and below-ground systems are highly coupled and alterations to vegetation structure, as can be caused by burning, must also be considered [76].

Other impacts of long-term use of prescribed burning on the peatland terrestrial habitat may include a lowered water table and lower pH [77], changes to soil water chemistry [78], and impacts on nutrient availability. Earlier research suggested that there may be long-term depletion of nitrogen (N), phosphorous and potassium [79] associated with managed burning. Subsequent studies concluded that these nutrient losses were replaced through precipitation [80–82], but more recent work has again suggested that N can be lost during prescribed burning [83]. The case of nutrient dynamics is, actually, a very interesting one as it nicely illustrates some of the complexities involved in categorizing fire effects as damaging or otherwise. Although losses of macronutrients may be easily perceived as a negative outcome of fire, nutrient deposition from atmospheric pollution has been one of the key drivers of degradation in blanket bog and heathland communities both within the UK and elsewhere in Europe [84]. Management activities that reduce nutrient availability in what are, by definition, low-nutrient systems may actually be beneficial for the recovery of key peatland species, such as *Sphagnum*, which are highly sensitive to increased nutrient loadings [85]. In this regard, the conclusions of Rosenburgh *et al.* [83], that large N inputs added via atmospheric pollution and subsequent soil N saturation can be alleviated by prescribed burning, are welcome.

Prescribed fire also has the potential for negative interactions with other land-management practices—especially, drainage and grazing (e.g. [86]). However, evidence for this is still rather patchy. For example, despite much research on the effects on heathland vegetation, evidence for vegetation succession pathways in response to combinations of burning, grazing and drainage in the UK uplands largely remains hypothetical particularly for peatlands (cf. [19,87]). Often studies are unable to untangle complex interacting disturbances: the paper by Bludnell & Holden [88], for instance, ascribes the loss of *Sphagnum* cover in a single case-study catchment to repeated severe wildfires but ascribes its lack of recovery to managed burning. However, they also acknowledge that subsequent nutrient and acid deposition from air pollution may also have been important. In aerial photographs, the area they studied (Lat: 53.853033, Long: −2.028975) shows extensive evidence of gullying and it is unclear whether this is related to a transition in the site's hydrological and ecological state following the compounded severe wildfires. When burning, grazing and drainage are carried out indiscriminately, these management practices are likely to be damaging to blanket bogs and may even lead to loss of habitat [87] and C. Re-wetting and restoration of drained peatlands is widely agreed upon as a management priority.

### (b) Understanding fire regimes: the importance of fire severity

While the effects of prescribed burning demand that we make trade-offs between different ecosystem services, there is growing evidence and consensus that severe, uncontrolled wildfires can have very serious consequences. Under drought conditions, wildfires can ignite peat layers causing smouldering peat fires and large emissions of C to the atmosphere [31,89]. Severe smouldering peat fires also have the potential to mobilize legacy pollutants in organic soils through volatilization or subsequent erosion (e.g. mercury [90]; lead [91]), which is of particular concern in heavily polluted peatlands in some areas of the UK (e.g. Peak District National Park, [92]). Even where peat itself is not ignited, severe wildfires show very different rates of ground biomass (moss, litter and duff) consumption compared with prescribed burns [24,25] and are potentially associated with changes to soil C dynamics [23]. Severe wildfires over organic soils can also produce a hard, hydrophobic bitumen surface that leads to increased run-off and changes to peatland hydrology with dramatic consequences for vegetation succession [68,93]. Severe wildfires have also been associated with lower rates of peat accumulation than unburnt areas [94], and the loss of *Sphagnum* cover [95]. We re-emphasize that the effects of severe wildfires should be separated from the outcomes of a carefully managed prescribed burn, and that the ecosystem outcomes of fire differ with wide variation in fire severity. Davies *et al.* [23,39] for instance, show that during managed fires consumption of the layer of moss and litter that overlies and protects the peat surface during burning is never more than ca. 20% of the pre-fire mass, and that bare-peat substrates following burning are the exception rather than the rule. Furthermore, Davies *et al.* [24,25] and Clay & Worrall [96] demonstrate that there can be very considerable variation in fire severity between and within individual wildfires. As we will highlight below, deliberately or accidentally confounding the effects of severe wildfires with those of low-severity prescribed burns (or even low-severity wildfires) can be very misleading.

### (c) Understanding fire regimes: the importance of fire frequency

The effects of fire vary both temporally and spatially with associated benefits and disbenefits depending on the scale one considers as well as the ecosystem services one is most interested in. Some changes are associated with the immediate aftermath of a fire (for example, changes to peat temperature regimes), while others (e.g. alterations to vegetation community structure) may only become apparent by taking a longer-term perspective. Fires vary in both their intensity and their severity, which is the result of spatial variation in vegetation/fuel structure and climate, temporal variation in fire weather (especially fuel moisture) between and within seasons and, in the case of prescribed burns, the expertise and care with which burns are managed. It is only by understanding the overall character of current and historic fire regimes (*sensu* [97]) that one can draw robust conclusions about the ecological effects of fire. In the UK, such information is conspicuous by its absence. A few trends have, however, been noted. Most managed burning in the UK is focused in core areas for grouse moor management in the Pennines, North York Moors and Grampian regions [18]. In these regions, fires on heather moorlands are recommended to be burnt on a rotation that would, very roughly, equate to a fire every 10–25 years [98]. That does not mean everywhere should be burnt that frequently. It has been suggested that such burning activity has been increasing within the Peak District [99] and nationally [18], while other, older research indicated that the use of fire as a management tool may be declining in Scotland [100]. Some of us have previously argued that such studies may be misleading and that less subjective methods are needed to map burning extent [24,25]. Crucially, none of the methods of estimating management history from aerial photographs used in previous studies have received any form of ground-truthing though more recently, Allen *et al.* [101] compared some of their results with estate managers' maps of burning activity. Nevertheless, the detailed national mapping study of Douglas *et al.* [18] is one of the best we have. They used visual inspection of aerial photographs to define areas of *Calluna*-dominated vegetation and mapped burning within such communities. Taking their estimates of a mean proportion of moorland burnt in the UK during the last 25 years (16.7%), the annual percentage area burned and mean fire rotation can roughly be estimated (*sensu* [102]). This results in 0.68% of moorland in Great Britain being burned per year (range on individual moors 0.04%–3.8%) and an average fire-return interval of 147 years (range on individual moors 26–2500 years)—assuming repeat burning within 25 years does not account for a significant area (the study by Allen *et al.* [101] suggests this is fair for at least some regions). Although these are very rough estimates, they suggest considerable heterogeneity in fire regimes across the British uplands, with the majority of sites probably experiencing fire-return intervals rather longer than the 10–20 years traditionally recommended for heather moorlands. This concurs with the results of Allen *et al.* [101] who showed that, for a case-study area within the Peak District, most burning followed recommended guidelines for fire frequency and fire size. The fire rotation values we estimate here are comparable with, or longer than, those associated with other peatland ecosystems where fire is a natural disturbance. For example, Vandvik *et al.* [14] summarized natural fire-return intervals in Norwegian boreal heaths and forests ranging between *ca* 100 and several thousand years (the latter being rare); Wieder *et al.* [103] documented fire-return intervals of 123 ± 26 years in black-spruce bogs in North American boreal forests. Yet, while fire frequency within landscapes is important, it is not the only variable of relevance in understanding the overall effect of fires. Rather, we need to quantify variation in the entire fire regime [97]. That not only includes fire frequency, but also fire intensity (rate of energy release during combustion), severity (immediate ecosystem effects such as vegetation consumption by the fire and sub-surface heating), extent, seasonality, and spatial and temporal variability in these attributes.

### (d) Monitoring peatland ecological condition in relation to fire

Despite the central role of fire in the ecology of UK peatland and moorland ecosystems, and the promotion of fire use for restoration of similar ecosystems in both southern [3] and northern (e.g. [20,21]) European countries, there is growing pressure to substantially reduce or even ban burning. Attention is often drawn to the fact that burning causes peatland ecosystems to be in ‘unfavourable condition’ [104]. However, this notion results from standardized assessment criteria that implicitly assume that fire only has damaging effects on peatlands and that, therefore, do not account for the fire ecology of our upland landscapes. The guidelines for Common Standards Monitoring practices [104] on peatlands in UK protected areas thus make it more-or-less impossible for burned sites to be classified as being in good condition (box 1), despite the potential ecological benefits of prescribed fire. Essentially, the presence or evidence of fire is a ‘fail’ criterion even when prescribed fire is part of an approved management agreement. As Yallop *et al.* [99] suggested, this inflates estimates of the impact of fire by assuming the whole site is affected by burning but also ignores any beneficial effects of fire. This monitoring therefore provides potentially misleading information, as large areas of peatland are recorded as degraded simply because they have experienced fire [104]. No attention is given to the nature of the burns or the character of the fire regime at the site.

Box 1. **Guidance from Common Standards Monitoring [104] for the condition of protected areas in the UK. Note that, in each case below, point two (no signs of burning or other disturbance) essentially covers all areas of blanket bog or wet heath dominated by *Sphagnum* and areas of blanket bog with abundant pleurocarpous and acrocarpous mosses. This means that not only can an area with *Sphagnum* not be in good condition if it shows any sign of being burnt, but areas of blanket bog not dominated by *Sphagnum* cannot be burnt either. Oddly, according to these standards it would not matter if a manager had burnt an area with a bare-peat substrate.**

With regard to burning, to be in ‘good condition’ the following conditions must be met in blanket bog habitats:

(1) There should be no observable signs of burning into the moss, liverwort or lichen layer or exposure of peat surface due to burning.
(2) There should be no signs of burning or other disturbance (e.g. mowing) in the following sensitive areas:
  — Ground with abundant and/or an almost continuous carpet of *Sphagnum*, other mosses, liverworts and/or lichens.
  — Areas with notably uneven structure, at a spatial scale of around 1 m^2^ or less. The unevenness should be the result of *Sphagnum* hummocks, lawns and hollows, or mixtures of well-developed cotton-grass tussocks and spreading bushes of dwarf shrubs.

For wet heath habitats to be in good condition, the following conditions must be met:

(1) There should be no observable signs of burning into the moss, liverwort or lichen layer or exposure of peat surface due to burning.
(2) There should be no signs of burning and other disturbance inside the boundaries of the ‘sensitive areas’ which includes ground with abundant, and/or an almost continuous carpet of *Sphagnum*, liverworts and/or lichens. This target should also be recorded if any evidence of this is found while walking between sample locations.

## 3. Generating informed, unbiased debate about the ecology of fire

### (a) Contextualization of fire research within academic literature and beyond

Prescribed fire provides an array of management benefits and challenges within a UK context that vary depending on the prioritized ecosystem services. Research has a key role in informing scientific, policy and public perceptions and debates on appropriate prescribed fire use. The interaction between research outcomes and society for a large part occurs through the public media. While science communication represents a difficult process of distilling technical research findings and complex messages into simplified media stories, effective and accurate communication is essential if appropriate land and fire management strategies are to be implemented. Unfortunately, the way in which research is presented in the media is not always unbiased, and research can be manipulated or misinterpreted by persons or groups that may have a pre-determined agenda. We emphasize the challenges of such debate through the discussion of recent case studies [17,18,99], some of which were highly publicized within the UK media. Through these case studies, we highlight how the scientific position can become skewed both within scientific publications themselves, and in their subsequent representation within the media.

### (b) Representation of fire in scientific publications

The context set by Yallop *et al.* [99] is a relatively balanced discussion of benefits and costs of managed fire, except for the fact that for some research areas they consider the evidence base to support their assertions was rather limited. This was (and still is) particularly true for the effects of fire on C sequestration. Yallop *et al.* [99] cite one paper where managed burning was shown to reduce C sequestration in peat bogs [65]. However, Garnett *et al.* [65] were unable to examine the effects across all replicates and treatments at their Moor House Hard Hill experiment and hence their experiment lost the power of the replicated experimental design. It is also evident that for much of Yallop *et al.*'s C-focused discussion there is a reliance on wildfire papers from boreal studies outside the UK (e.g. [94,105]). This is undoubtedly a consequence of the lack of local evidence in this research area, but there is no clear acknowledgement of this carbon knowledge gap or how it impacts on the scientific debate being put forward. This seems a relatively important point given the way the media picked up on their study, choosing to concentrate on burning impacts on peatland C emissions rather than the mapping exercise the paper was concerned with (see section Representation of science within the media).

The study of Douglas *et al.* [18] sets a context in which the effects of fire on the natural environment in general are primarily negative. While initial mention is made of potential positive benefits of burning, the authors go on to question the widely accepted benefits of prescribed fire for wildfire hazard reduction [3,4,106], citing Altangerel & Kull [107] and suggesting that ‘the benefits and disbenefits [are] debated’. In reality, Altangerel & Kull [107] themselves conclude that ‘differences in how people frame the risks of prescribed burning, the certainty of its outcomes and what values they evoke in order to justify their views do not necessarily arise from divergent priorities about nature, people or assets, but instead from contrasting views about whether humans or nature are voluntarily or involuntarily exposed to wildfire risk’. Thus, the debate is not so much about the effectiveness of fire as a tool but rather about philosophical and societal responses to its use. Interestingly, Altangerel & Kull [107] point out that both citizen groups in favour of and those against prescribed burning tend to selectively frame their arguments to build support for their views.

Douglas *et al.* [18] refer to ‘increasing evidence of negative environmental impacts of burning’ across ‘a range of systems’. A number of papers or reports are cited to suggest negative impacts of fire on soil erosion [108], nutrient cycling and soil hydrology [109], water quality [110], air pollution [111], and *Sphagnum* plants [77]. However, this focuses on the short-term impacts of an individual fire rather than long-term ecosystem dynamics across the fire cycle. It also fails to recognize the complex messages from each of these studies in which clear benefits of fire management could also be highlighted. Cawson *et al.* [108] showed that catchment-scale studies usually report minimal impacts of prescribed burning on post-fire run-off and erosion from mineral soils. They stressed the importance of understanding how fire characteristics affect post-fire run-off and erosion, as fire regimes can be manipulated to reduce potential erosion and water quality impacts. Neary *et al.* [109] reviewed the direct effects of fire on below-ground systems (mostly mineral soils) and described them as a function of burn severity, which integrates aboveground fuel loading (live and dead), soil moisture, duration of the burn and post-fire soil temperatures. Tian *et al.* [111] assessed atmospheric emissions of prescribed burns and showed that corresponding air quality impacts can be mitigated by forest management practices. For example, where prescribed burning is less frequent, increasingly more fuel is burnt in each fire, leading to higher emissions and greater air quality impacts per fire. Brown *et al.* [77] is cited to support the argument that fire has a negative impact on *Sphagnum* plants, but the focus of this report is on aquatic ecosystems and catchment hydrology; the authors make no direct observations of fire's impact on *Sphagnum* itself and this assertion is in conflict with the results of Lee *et al.* [40]. Further, Douglas *et al.* [18] contextualize their research within the debate about the relationship between fire and peatland carbon dynamics. Despite this, many of their assertions are not currently supported by scientific consensus, which is partly demonstrated by their reliance upon unpublished or non-peer reviewed reports (e.g. [77,112]), hereafter termed grey literature. This highlights an important issue: where the fire evidence base is weak, grey literature can often be the only source of evidence. While grey literature is used in scientific evidence reviews and meta-analyses to counter publication bias [113] one aspect of using it authors should perhaps attempt to avoid, is the tendency to cite without critical assessment. For example, the report by Brown *et al.* [77] did not include any fire ecology measures (e.g. severity) and lacked a detailed description of the statistical models used in the analyses. The experimental design was fairly complex, including fire chronosequences and different sampling intensities across certain sites. A lack of statistical methodology makes scientific evaluation of findings problematic [114]. Unfortunately, a critical and balanced assessment was, through no direct fault of the report's authors, also lacking from the resulting media reports that followed publication of the Brown *et al.* [77] report (table 1). Although a substantial proportion of the results presented in Brown *et al.* [77] have now been published in peer-reviewed journals, it would have been preferable to have published a report that could be scrutinized in more detail and to have subjected findings to peer review before releasing the summary report.

**Table 1.** A selection of recent mainstream media (i.e. newspapers, network television websites and scientific magazines) coverage associated with scientific papers and reports concerning the use of fire on UK peatlands. We did not consider articles from, for example, non-governmental organization membership magazines or publications associated with particular land-use sectors. The main quote is the first paragraph of the article. While many of the articles provided some balance by reporting the opinions of a range of stakeholders including those involved in the game industry, few provided an opinion from a non-associated scientist or reflected the uncertainties involved in assessing the complex effects of prescribed burning.

media title	main quote	media reference	associated paper
‘Grouse-shooting popularity boosts global warming’	‘The ‘glorious 12^th^’ falls this weekend. It's the start of the UK's grouse-shooting season, attracting the rich and famous from around the world. But the country will be getting a bigger bang than it bargained for. Attempts to breed more grouse on the moors to meet rising demand are boosting the UK's contribution to global warming.’	Pearce [115]	Yallop *et al.* [99]
‘Cut heather burning for sake of the environment’	‘Ember study suggests muirburning degrades upland moorland, its fauna and flora.’	Amos [116]	Brown *et al.* [77]
‘Burning debate reignited’	‘Heather burning on moorland has ‘significant negative impacts’ on natural habitats, according to a study by academics, claims which have been countered this week by the Moorland Association.’	Barnett [117]	Brown *et al.* [77]
‘‘Amazon of UK’ being destroyed for grouse shooting’	‘Managing moorlands so that more birds can be reared for lucrative shooting parties is adding to climate change by destroying layers of peat and releasing large quantities of stored carbon dioxide into the atmosphere.’	Brown [118]	Brown *et al.* [77]
‘Peatlands burn as gamekeepers create landscape fit for grouse-shooting’	‘They are home to a diverse range of wildlife and up to 8000 years old. And, according to a damning analysis by an independent government advisory body, the UK's upland peat bogs are facing a sustained threat from the shooting classes' desire to bag grouse.’	Doward [16]	Douglas *et al.* [18]
‘Burn moor, or less?’	An authoritative study has revealed the environmental effects of moorland burning. The Effects of Moorland Burning on the Ecohydrology of River basins project (EMBER), adds to a debate over grouse moor management.	Hart [119]	Brown *et al.* [77]
‘Why we should rewild the British uplands’	‘The upland environment covers a third of Britain. It is a cherished landscape, close to the hearts of most of us. Much of this landscape is within National Parks celebrated for their ‘natural beauty’. Yet, for the most part, whilst they are beautiful, they are a far way from a natural environment. They are overgrazed sheep pastures and burnt grouse moors.’	Manighetti [120]	Brown *et al.* [77]
‘Feeling the heat from peatland vegetation burning’	‘There are more than 1.5 million hectares of peatlands in the UK, covering 17.2 per cent of the land surface. Upland moorlands face a range of management pressures in the UK, and recent research shows vegetation burning in peatlands has altered the biodiversity of their rivers.’	Ramchunder [54]	Brown *et al.* [77]
‘Burning of heather ‘damaging peatlands and rivers’’	‘The tradition of burning heather on sporting estates causes significant environmental damage to both peatlands and nearby rivers, according to a new authoritative scientific study.’	Ross [121]	Brown *et al.* [77]
‘Regular burning of English upland peatlands must stop: new study shows damage much worse than thought’	‘Every big scientific project needs a good acronym these days and the Leeds University team hits the spot with EMBER—Effects of Moorland Burning Ecohydrology of River basins. And in line with the acronym, the results show that the damage that burning heather has on wildlife, climate change and the environment is far worse than previously thought, and more wide ranging—water run-off from burned peat harms aquatic life in the rivers that spring from these uplands. In short, managed burning has a profound impact on the life support systems of the peatlands in our hills.’	Watts [122]	Brown *et al.* [77]
‘Moorland burning ‘threatens protected landscape’’	‘It is a traditional tactic used over the decades to regenerate the stunning moorland landscapes that attract thousands of visitors to the region every year but an old debate over its contribution to wildlife conservation has been re-ignited’.	Barnett [123]	Douglas *et al.* [18]
‘Shooting industry must stop putting strain on countryside, says RSPB chief’	‘More than 50 million game birds a year are being released for shooting, putting increasing strain on native wild birds and the ecology of the UK's countryside, landowners will be warned on Friday.’	Davies [124]	Douglas *et al.* [18]
‘Britain's ‘protected’ moorlands go up in flame’	‘A new study led by RSPB shows that more than half of Britain's most precious upland moors are suffering from burning—widely used to increase the numbers of red grouse available for recreational shooting.’	The Ecologist [125]	Douglas *et al.* [18]
‘Moorland report criticises heather burning’	‘The practice of scouring moorland by burning off heather has left many conservation areas in Scotland in a poor condition, a charity has said’	Harrison [126]	Douglas *et al.* [18]
‘Protest against grouse shooting on Ilkley Moor’	‘A protest will take place this morning against controversial grouse shooting on Ilkley Moor. The event at Bradford City Hall coincides with the opening of the burning season, when moorlands are set on fire to increase game bird numbers for shooting.’	ITV [127]	n.a.

Brown *et al.* [17] reviewed the impacts of fire on the hydrology, biogeochemistry and ecology of peatland river systems, and gave a relatively thorough overview of the limited existing evidence of the changes that burning can induce in hydrological and aquatic systems. In some places, however, their discussion appears to restate popularly held but unsupported assumptions and to rely heavily on unpublished material. For instance, in the section of their paper concerning fire effects on terrestrial vegetation, they state ‘Burning is considered particularly detrimental to peat-forming *Sphagnum* species’. Although they do acknowledge that there is contradictory evidence in the scientific literature ‘from a small number of experimental burning plots’ [40], the only citation to support the initial assertion is a report by the Royal Society for the Protection of Birds (RSPB; [128]) that has not been formally published or, to our knowledge, peer reviewed. Grant *et al.* [128] actually state fairly clearly that *Sphagnum* species are only ‘considered’ to be fire sensitive and that the true picture is likely to be much more complex. In reality, *Sphagnum* flammability and sensitivity to burning is likely to be species-specific and to vary between ‘hummock’ and ‘hollow’ species [129]. There is considerable evidence from other systems that common species, such as *S. fuscum* and *S. cappillifolium*, are relatively resilient to fire [130].

Later, Brown *et al.* [17] point to government guidelines that ‘recommend against burning into living moss layers’ but then comment that ‘this level of control is not always achievable’. Notwithstanding the fact that the fuel moisture content of moss layers during the legal burning period are often high enough to make deep combustion physically impossible in all but the most severe droughts [22], there is good evidence that moss consumption during prescribed burns is very limited and that exposure of bare peat is rare [39]. Where Brown *et al.* [17] suggest burning leads to peat exposure, their citations relate to the outcomes of severe wildfires rather than prescribed burns. They, therefore, make the common mistake of conflating fire intensity and degree of control with fire severity, when in reality the link between intensity and severity is complex [24,25,39]. While Brown *et al.* [17] are right to point out that burn management is often far from perfect, we still have very little data on how management practices vary across the UK and again need to realize that the issue in question is not as simple as burnt/unburnt but rather how ecosystem changes scale across variation in fire regimes (i.e. frequency, extent, intensity, severity, seasonality and variability in these) and the fire cycle.

Finally, Brown *et al.* [17] rightly point out that much of our knowledge comes from a single long-term experimental study site (the Hard Hill burning/grazing experiment in Cumbria, UK), but then they seek to suggest (again on the basis of an unpublished RSPB report) that the results from that location are not generalizable as the fires are ‘extremely controlled’; despite the fact that the use of controlled fire is precisely the aim of prescribed burning. As far as we are aware, no data have actually been published on prescribed burning practices at Hard Hill or the behaviour of the fires burnt there. Furthermore, the inference that at all other sites fire conditions are not ‘extremely controlled’ would perhaps imply that moorland managers are either not very good at, or do not care about, adequate fire control. In the above examples, we are not trying to suggest that Brown *et al.* [17] are being deliberately misleading, rather that tone, precision of language and an acknowledgement of complexity is very important. There are substantial opportunities for misunderstanding which could result in it being deemed that the skill, intentions or understanding of particular stakeholder groups is under question. In reality, it is in the interests of managers to ensure fires do not grow too large or intense such that they would destroy the habitat matrix grouse require, or such that they put *Calluna* regeneration at risk. Further data on variation in prescribed burning practice (e.g. average fire size distribution, orientation along slopes, spatial distribution within landscapes in relation to sensitive areas such as scree or riparian zones) would be welcome. The results of Allen *et al.* [101] show that, at least in their study area, practice meets existing guidelines and fires are well controlled.

In summary, these three case studies create an unbalanced tone in which the outcomes of fire are presented as generally negative. Of course, it is clear that episodic disturbances induce significant changes in a range of environmental parameters, and that variation in disturbance regimes can drive changes in ecological structure and function. Whether these changes/differences are positive, negative or of no consequence is likely to depend upon the spatial and temporal scales, and ecosystem services, one chooses to focus on. A key issue with all three case studies is that some of the evidence upon which they base their assertions is limited or incomplete, and following the citation trail often reveals insufficiently critical reliance upon either unpublished reports or a simplistic (mis)interpretation of complex scientific findings.

### (c) Representation of science within the media

The use of fire as a management tool within the media often appears to similarly lack nuance. For example, a recent newspaper article by Monbiot [131], provocatively titled ‘Meet the conservationists who believe that burning is good for wildlife’ with the sub-heading ‘Our national park authorities are vandals and fabulists, inflicting mass destruction on wildlife and habitats, then calling it conservation’, emerged while this paper was in initial review and is only the latest in a line of contributions where the tone is not conducive to inclusive, balanced debate. With regard to the papers we assessed above, for both Yallop *et al.* [99] and Douglas *et al.* [18], the subsequent reporting in the media from associated press releases unfortunately did not focus on the strengths of their research findings. Instead, the press releases used the papers as an opportunity to make tangential and provocative inferences about associated issues. The almost hysterical headlines of some news items were particularly striking (table 1). The tone of many of these articles was staunchly anti-burning and focused on purported negative impacts of fire, even if this bore little relationship to the studies' actual focus. Many media articles concerning managed burning appear to be highly biased. For example, in the case of Pearce [115], the focus, C losses as a result of burning, was not measured in the work reported by the scientific paper on which it was reporting [99], and the quotes and narrative it contained were highly speculative. In the case of Doward [16], the news item appeared to suggest that research conflicting with the main anti-fire narrative was influenced by its funding source (The Game and Wildlife Conservation Trust, an organization that has a large number of members active in game management or hunting, but which is also a well-regarded research and conservation charity).

Unfortunately, as scientists we often have little control over the representation of our research within the media. Others have noted how the characteristics of scientific claims change between scholarly writing and non-specialist audiences [132], and this is likely to remain a problem when journalists, unlike scientists, routinely refuse to allow pre-publication review of their articles even by those whose research they are covering. Despite this, the tone with which scientific output is covered in the media can be moderated through careful positioning of the research within the academic literature and in any associated press releases. We have no access to press releases from the publication of Yallop *et al.* [99], but in our view the three different press releases associated with the Douglas *et al.* [18] paper [133–135] are not a direct reflection of the key findings of the paper: while listing the extent of moorland burning found in the scientific paper, comments in the press releases are primarily made regarding the ‘damaging’ effect of fire. This is perhaps of little surprise given that the RSPB is in frequent conflict with the UK land-management community over a range of issues, including the ethics of driven grouse shooting and the persecution of raptors [136]. Individuals closely associated with the RSPB have made unambiguous calls for burning to be banned [137]. We, therefore, suspect that much of the contextualization in recent fire-related studies stems less from evidence of the environmental effects of managed burning and more from attitudes towards the forms of land ownership and other management practices associated with burning in the UK. There are undoubtedly systemic issues associated with some aspects of grouse moor management in the UK (e.g. [136]), and it has been previously argued that fire management practices that are focused solely on production of grouse, and justified on the basis of tradition alone, are unlikely to provide ecologically resilient, multifunctional upland landscapes [8]. While the cultural history of fire use can be an important consideration in determining fire management policy [138,139], it should certainly not be used as justification for the continuation of unsustainable practices. Here the picture becomes more complex as perceptions of sustainability depend upon the ecosystem services a particular group or individual prioritizes and there are inevitable trade-offs between different services [140].

There are a wide range of views on issues regarding the socio-economics and ethics of private estate ownership and driven grouse shooting in the UK, both within the research community at large and among the authors here. Effective communication and understanding between different groups currently seems to be minimal. Studies suggesting that some land managers believe they have access to ‘special’ knowledge regarding moorland that others cannot comprehend [141] are thus concerning. However, so too is the fact that conservationists often seem unable to make objective interpretations of individual ecological management practices, such as prescribed burning, independent of the wider moorland management context. There can often be a complex relationship with managed fire even within a single organization. In our own research, we have experienced managers in one region not prepared to contemplate even a single research burn on a bog, while those from the same organization but based an hour up the road have actively sought us out to trial burning on similar sites. Furthermore, RSPB research has shown the value of prescribed fire as a tool to promote woodland expansion at forest–moorland edges and to manage Capercaillie (*Tetrao urogallus* Linnaeus, 1758) habitat [142]. By campaigning so strongly on the presumed negative effects of burning on peatland ecosystems the RSPB thus risks undermining the ability of its own managers to use fire as an ecological tool. Organizations like the RSPB, which have to balance ecological campaigning and management roles, often face the challenge of aligning local management needs with dominant narratives or ‘party lines’. It would be preferable if ecological knowledge were allowed to determine attitudes rather than vice versa. We recognize that individuals are at liberty to form their own opinions on subjective issues like the aesthetics of certain landscapes or the ethics of hunting, but as environmental scientists we have a duty to ensure we do not conflate opinion with evidence and to acknowledge where we lack knowledge. The problem with the tone of the current debate in the media was neatly summarized by Thorp [143]: ‘I was struck by what a waste of time these exchanges were, as no one is going to trot out anything but their safest party line on these occasions. In my view, this type of exchange only serves to feed sensation, deepen the trenches and sell publications/increase ratings.’

### (d) Assessing how science communication affects perceptions

To determine how non-specialists' perceptions of fire are influenced by differences in reporting in academic and public media, we distributed one of the following to each of six separate groups of six to seven senior undergraduate and graduate students of restoration ecology at The Ohio State University (USA): the results or discussion sections of Douglas *et al.* [18]; an associated RSPB press release [133]; and subsequent media coverage [16,125]. The material extracted from Douglas *et al.* [18] was modified to remove the citations so it was not immediately apparent what kind of publication each reading came from. Each group of students was asked to come to a consensus about what they perceived to be the two key research findings of their reading. Responses from those reading the results section correctly concluded the key findings were that burning was increasing and that it was strongly associated with protected areas. This was in contrast with responses from those reading the discussion, press release and newspaper articles, who concluded that key findings were that burning took place in protected areas and that burning was damaging to peatland ecosystems. The difference in the groups' perceptions demonstrate Douglas *et al.*'s own discussion of their findings, and the associated outreach and media coverage gives the impression that the paper focused on the environmental and ecological effects of burning. In reality, the work described the spatial distribution of burning and short-term temporal trends in fire; the results of which have been questioned [24,25].

If we are to debate the use of fire as a management tool, it is essential that authors ensure that the press releases associated with their findings accurately reflect the content of their research as well as the uncertainty associated with ongoing research questions. At the same time, it is also essential that journalists reporting on this clearly contentious topic do not just rely on the content of press releases from campaigning organizations but verify facts by reading the actual paper and consulting with an independent academic expert not involved in the study. Journalists reporting on scientific findings need to decide whether their duty is to report science or further their own or others' agendas. Journalists should preferably adopt a neutral tone and make a clear distinction between research reporting and opinion pieces.

## 4. Priorities for future research

Fire as a management tool is carried out at the landscape scale and induces ecological processes that span from minutes to decades following the burn. Most research relies on small plots of 1 to tens of meters and monitoring might, at best, extend for a couple of years following the fire. The only UK site where long-term evidence is available on peatland burning is Moor House in the Pennines. Even these experimental plots are not at a landscape scale (900 m^2^ [144]) and the fire rotations are unlikely to be applied in real situations as recommendations stipulate longer rotations in peatlands (see Muirburn Code; [98]). Alternatives are to take chronosequence or catchment comparison type approaches as these are often the only way to approach questions regarding longer-term fire effects in the absence of replicated experiments. Unfortunately, such studies are replete with assumptions, for instance that catchments would have similar physical, chemical and hydrological characteristics in the absence of burning. They can also have difficulty in ascribing causality, particularly where past and present management regimes cannot be adequately documented. For instance, past wildfire history may also be an important component of the fire regime. Developing an integrated, holistic understanding of the effect of variation in fire regimes on peatland ecosystems is likely to require a combination of study types and a multidisciplinary approach including land managers, ecologists, hydrologists, fire scientists, sociologists and economists (Roos *et al.* [145]). Coordinated, distributed experiments [146] across different peatland ecosystems perhaps also hold promise if our aim is to try and develop more generalizable knowledge regarding fire effects on peatlands. Much knowledge also exists elsewhere in northwest Europe, where many peatland ecosystems have similar vegetation and management histories (e.g. [12]). Limited funding for peatland research means that research groups often seem to be in competition with each other. This has had an effect on research quality as groups with widely differing backgrounds and expertise (e.g. hydrologists, plant ecologists, avian biologists, carbon scientists, fire scientists) try to be all things to all people, leading to inevitable gaps in knowledge and understanding that subsequently surface in methodologies and interpretations. A good example of this can be seen in the recent exchange between Douglas *et al.* [147] and Davies *et al.* [24,25]. Here, the reasons for the misinterpretation of the results of moderate-resolution imaging spectroradiometer (MODIS) fire detections by Douglas *et al.* [18] was revealed in their subsequent response [147], as it became apparent they had confused the concepts of ‘burn area’ (i.e. the total area burnt by a fire) with ‘fire front area’ (the area of actual flaming combustion at any one point in time). We agree with the proposition in Brown *et al.* [17] that more integrated working between researchers is needed, and that catchment-scale manipulations and networks of long-term experimental burn sites are urgently required. Working in partnership with land managers, fire professionals and other non-academic stakeholders to co-produce knowledge is another approach to extend the spatial and temporal range of data collection, incorporate local knowledge and build trust [148,149].

Recent reviews (e.g. [35]) have drawn attention to the very substantial knowledge gaps that remain with regard to the effects of fire on peatland ecosystems. We do not dispute the fact that fire causes a range of ecological and environmental changes—some of which are less welcome than others and have a mixture of costs and benefits. There is, however, very considerable uncertainty, and knowledge is missing in several key areas. Ongoing research in the UK is certainly not being helped by the fact that several studies seem to be operating in a vacuum where understanding from wildland fire science and peatland ecology more generally is missing and leading to methodological and interpretational errors. In particular here is the argument from wildland fire scientists in the USA (e.g. [150,151]) and Mediterranean (e.g. [152,153]) that fire exclusion (or the ‘over-suppression paradigm’) allows fuels to accumulate and ultimately increases fire intensity and burn severity. This hypothesis has not yet been tested in the UK context but is often touted as a benefit of managed burning. Indeed even a baseline assessment of fuel load and continuity would be a welcome start.

Whether or not current land-management priorities, burning regimes and other practices are ecologically sustainable, or morally justifiable, in the context of social and environmental change are questions that still require much further study and debate. There is currently little scientific consensus either way, with often contradictory results on the effects of fire on DOC concentrations in moorland water [57] and gaseous C emissions from peat soils where, again, the majority of the evidence is from Moor House (e.g. [154]). Some results, such as the finding that burning benefits at least some *Sphagnum* species [40,155] directly challenge current perceptions and require further study. Brown *et al.* [17] were right to point out that too much of our knowledge comes from a small number of sites and that experimental treatments may not be representative of the variety of management practices on the ground. Larger catchment-scale comparisons of the type completed in Brown *et al.* [77] should be welcomed, though they also have their issues as they make the implicit assumption of similar long-term historical land use and similar underlying abiotic conditions (something the results of [83] suggest is unlikely).

We argue here that the following important factoids are not verified. They require further study and should not be perpetuated in discussions until they are formally addressed:

— That regular burning alone increases *Calluna* dominance. Areas associated with burning tend to have greater *Calluna* cover but managers do not distribute their effort randomly across landscapes and it is unclear if burning is the result or cause of increased *Calluna* cover. Time scale is also important. Indeed, not burning vegetation with a substantive *Calluna* component will increase its dominance at least over a 90-year period, a time range close to the natural historic fire-return interval of 120–200 years [40].
— That fire kills or significantly damages *Sphagnum*. We need to quantify species responses to fire and to understand the importance of variation in burn severities and frequencies. *Sphagnum* species display micro-habitat differences (hummock, hollow, pool and lawn) and it is likely that micro-habitats will respond to burning differently given their distinct topography and moisture regimes. We also need to know whether burning limits *Sphagnum* recovery during peatland restoration and if so, under what fire regimes?
— That peatlands are particularly sensitive sites with regard to fire. Northern peatlands elsewhere in the world, notably within boreal regions, can show remarkable ecohydrological resilience to burning [156,157]. Interactions with drainage can, however, induce significant changes in this regard [158]. Such findings have received little attention in the context of UK peatland management.
— That managed burning helps protect against future wildfires, minimizing fire likelihood and burn severity. How does managed burning affect landscape-scale patterns in flammability; does it reduce the frequency or burn severity of wildfires? How many wildfires actually result from managed burning? In other words, how do wildfire and managed fire regimes interact?
— That fire alone can contribute to peatland degradation. At what frequencies or severities is this true, if at all? How can we separate the confounded effects of drainage, grazing, acidification and nutrient deposition? Unlike wildfires, managed burns appear rarely to leave areas of peat exposed, but might this vary according to fire frequency? Over what spatial and temporal scales should degradation be defined?

## 5. Conclusion

Fire is a valued and integral component of the ecosystem manager's tool kit capable of being used as well as abused in a multiplicity of different ways. Throughout Europe, managers, ecologists and conservationists value prescribed burning as a tool to protect and restore globally rare heathland and moorland ecosystems and there is a growing body of scientific literature to inform best practice. Much of this knowledge comes from research in the UK and it is ironic that while the public debate here has shifted strongly against the use of fire, scientists in other countries are using this evidence to promote the reintroduction of burning. Further scientific evidence is urgently needed on the benefits and costs of differing fire regimes for peatland and moorland ecosystem services. Such assessments need to focus on the landscape scale and on elucidating trends over the entire fire rotation rather than just looking at the short-term outcomes of single burns that are a pulse disturbance with obvious negative outcomes for particular metrics. Until integrated evidence is available, all scientists should be concerned when potentially interesting and informative research is used as a forum to propagate what amounts to hearsay or to promote political agendas. The use of press releases to publicize a particular point of view when the actual scientific evidence from a study is incomplete or unrelated should be discouraged.

In the absence of sound evidence and consensus, it is vital that managers and scientists adopt an ‘adaptive’ approach to decision making [159]. Core principles of adaptive management include the need to monitor and learn from management actions, to keep an open mind until the evidence is settled and consensus reached, and to involve all stakeholders and viewpoints in decision making. Managing for a single ecosystem service, be that traditional burning practices for game production or banning burning to try and reduce the colour of drinking water, is unlikely to be sustainable if the wider impacts of management regimes are not considered [140]. It is vitally important for both scientists and journalists to ensure objective outreach and reporting on this ongoing and contentious debate as trust between stakeholders risks reaching rock-bottom.

Restoring resilient peatland ecosystems that protect existing carbon stocks and function as a carbon sink is a priority for the UK and we welcome initiatives such as Scottish Natural Heritage's Peatland Plan [160]. What is clear to us is that approaches to science and science communication that ignore complexity, seek to propagate agendas and alienate stakeholder groups on either side of the debate are not doing anyone a favour in the long term. A narrow ‘bounded rationality’ that bases decisions on evidence from a selective perspective instead of a holistic one is liable to lead to policy failure, as Busenberg [150] argues was the case for US fire policy. Prescribed burning is a potential tool for peatland management and restoration along with other techniques such as grazing, cutting or ditch-blocking. Like all ecological tools, fire can be used well or poorly and will not be suitable in all situations. We are certainly not arguing that across the UK the status quo necessarily represents best practice or that it will deliver resilient peatland ecosystems. However, if we want to retain moorlands and peatlands as one part of a diversity of upland landscape structures, fire will need to be part of their management. Although managers seem to mostly follow current recommended guidelines on burning [161], traditional approaches to managed burning have room for improvement but do deliver important conservation benefits [8,19]. Our objective should be to use fire as one tool in management that aims to produce structurally diverse upland landscapes that protect a range of ecosystem functions. The conversation needs to move away from unhelpful hyperbole about banning part of the ecosystem manager's toolkit and focus on learning how to use it well. This could include better technical training in fire use, certification for fire users, explicit integration of knowledge regarding relationships between fire behaviour and fire effects, and an increased emphasis on monitoring and compliance. Such changes would be a first step to facilitating more precise and targeted fire use that maximizes benefits, minimizes detrimental environmental impacts and builds trust between stakeholders.
